# Shp2 Plays a Critical Role in IL-6-Induced EMT in Breast Cancer Cells

**DOI:** 10.3390/ijms18020395

**Published:** 2017-02-13

**Authors:** Xuan Sun, Jie Zhang, Zhiyong Wang, Wei Ji, Ran Tian, Fei Zhang, Ruifang Niu

**Affiliations:** 1 Public Laboratory, Tianjin Medical University Cancer Institute and Hospital, National Clinical Research Center for Cancer, Tianjin 300060, China; sunxuandoctor@163.com (X.S.); zhangjiehmy@163.com (J.Z.); wzy7848354@hotmail.com (Z.W.); jiwei217@126.com (W.J.); tianrants1985@163.com (R.T.); 2 Key Laboratory of Cancer Prevention and Therapy, Tianjin Medical University Cancer Institute and Hospital, Tianjin 300060, China; 3 Tianjin’s Clinical Research Center for Cancer, Tianjin Medical University Cancer Institute and Hospital, Tianjin 300060, China; 4 Key Laboratory of Breast Cancer Prevention and Therapy, Tianjin Medical University, Ministry of Education, Tianjin 300060, China; 5 Cambridge-Suda Genome Research Center; Soochow University, Suzhou 215123, China

**Keywords:** Shp2, EMT, IL-6, invasion, breast cancer

## Abstract

Accumulative evidence demonstrates that the protein tyrosine phosphatase Shp2 functions as a powerful tumor promoter in many types of cancers. Abnormal expression of Shp2 has been implicated in many human malignancies. Overexpression of Shp2 in cancer tissues is correlated with cancer metastasis, resistance to targeted therapy, and poor prognosis. The well-known function of Shp2 is its positive role in regulating cellular signaling initiated by growth factors and cytokines, including interleukin-6 (IL-6). Several recent studies have shown that Shp2 is required for epithelial-mesenchymal transition (EMT), triggered by growth factors. However, whether Shp2 is involved in IL-6-signaling-promoted breast cancer EMT and progression, remains undefined. In this study, we showed that exogenous and endogenous IL-6 can enhance breast cancer invasion and migration, through the promotion of EMT. IL-6 also induces the activation of Erk1/2 and the phosphorylation of Shp2. Knockdown of Shp2 attenuated the IL-6-induced downregulation of E-cadherin, as well as IL-6-promoted cell migration and invasion. Moreover, by using Shp2 phosphatase mutants, phosphor-tyrosine mimicking, and deficiency mutants, we provided evidence that the phosphatase activity of Shp2 and its tyrosine phosphorylation, are necessary for the IL-6-induced downregulation of E-cadherin and the phosphorylation of Erk1/2. Our findings uncover an important function that links Shp2 to IL-6-promoted breast cancer progression.

## 1. Introduction

The SH2 (Src homology region 2) domain-containing protein tyrosine phosphatase (Shp2), which is also known as protein tyrosine phosphatase 2C (PTP-2C) or protein tyrosine phosphatase 1D (PTP-1D), is a non-receptor protein tyrosine phosphatase, encoded by the human *ptpn11* gene [1]. The structure of Shp2 consists of two tandem SH2 domains in the N-terminal, a protein tyrosine phosphatase (PTP) domain, and a C-terminal region [2]. In a quiescent state, the phosphatase activity of Shp2 is inhibited by SH2 domains, which bind to the PTP domain and result in inactive conformation. The binding of phosphotyrosyl residues to SH2 domains relieves this autoinhibitory effect and activates the phosphatase function of Shp2 [3]. In addition, the tyrosine phosphorylation of Shp2 in its C-terminal region (Tyr542 and Tyr580), regulates the phosphatase activity and changes its substrate specificity [4]. The phosphatase ability of Shp2 can dephosphorylate large quantities of important signal molecules. Aside from being an enzyme, Shp2 also functions as an adaptor protein, by nucleating multiple signaling proteins. A well-known function of Shp2 is its ability to promote maximal activation of the Ras/mitogen-activated protein kinases (Ras/MAPK) signal pathway, initiated by growth factors, including epidermal growth factor (EGF), fibroblast growth factor (FGF), insulinlike growth factors (IGF) and platelet derived growth factor (PDGF) [5]. Intensive studies have proposed that Shp2 also mediates cellular signaling in response to cytokines, hormones, and cellular stresses in phosphatase-dependent and -independent mechanisms [6]. The abnormal expression of Shp2, or a Shp2 mutation, is associated with many human diseases, including Noonan syndrome, LEOPARD syndrome, leukemia, and solid tumors [7,8]. However, the way in which Shp2 affects these diseases remains unclear.

Although phosphatases are strong potential tumor suppressors, accumulative evidence demonstrates that Shp2 functions as a powerful tumor promoter in many types of cancers [9,10,11]. Somatic dominant-active mutations of Shp2 are frequently detected in human hematological malignancies [12]. Although Shp2 mutations in solid tumors are less frequently observed than those in leukemia, the overexpression of Shp2 is common in many types of carcinoma, including breast, lung, colon, pancreatic, and prostate cancer [13,14,15]. An elevated Shp2 level in breast cancer tissues is correlated with cancer metastasis, resistance to targeted therapy, and poor prognosis. Intensive studies that conducted Shp2 loss- and gain-of-function analyses, have suggested that Shp2 is a critical modulator for cancer cell survival, proliferation, invasion, cytoskeleton reorganization, angiogenesis, and drug resistance [16,17]. Moreover, several recent studies have demonstrated that Shp2 is an essential promoter of epithelial–mesenchymal transition (EMT), triggered by growth factors, including EGF, PDGF, and transforming growth factor-beta (TGF-β) [18,19,20]. However, the underlying mechanism by which Shp2 regulates EMT and promotes cancer progression, remains largely undefined.

Apart from positively mediating receptor tyrosine kinase signaling, Shp2 also regulates transducing signals initiated by cytokines, including interleukin-6 (IL-6) [21]. Elevated serum IL-6 levels and increased activation of the IL-6 signaling pathway, have been observed in many human malignancies, and are associated with cancer initiation and progression [22,23]. In addition, a previous study reported that IL-6 is a potent inducer of EMT, especially in breast cancer cells [24]. However, whether Shp2 is involved in IL-6-signaling-promoted breast cancer EMT and progression, remains uncertain. In this study, an IL-6-induced EMT model of breast cancer was established by the overexpression of IL-6 in T47D cells. IL-6 promoted EMT through the increased activation of Erk1/2 and the phosphorylation of Shp2. Knockdown of Shp2 attenuated the IL-6-induced downregulation of E-cadherin, as well as IL-6-promoted cell migration and invasion. By using Shp2 phosphatase mutants, phosphor–tyrosine mimicking, and deficiency mutants, we provided evidence that the phosphatase activity of Shp2 and its tyrosine phosphorylation, are necessary for the IL-6-induced downregulation of E-cadherin and the phosphorylation of Erk1/2. Our findings uncover an important function that links Shp2 to IL-6-promoted breast cancer progression.

## 2. Results

### 2.1. Exogenous IL-6 Induces EMT and Increases Cell Migration In Vitro

**Figure 1:**
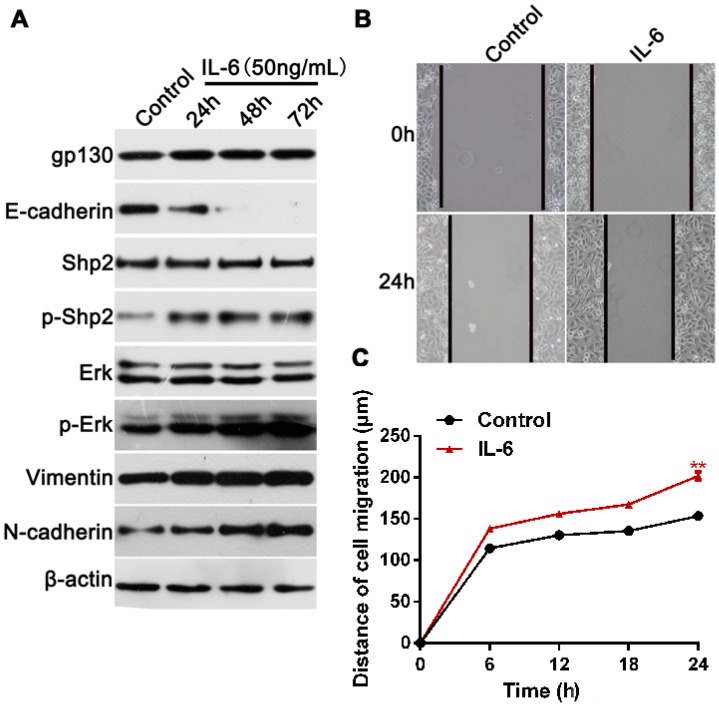
Exogenous IL-6 treatment induces a significant increase in cell migration ability in vitro. (**A**) Western blot analysis of E-cadherin, N-cadherin, Vimentin, Shp2, phosphorylated Shp2, Erk1/2, phosphorylated Erk1/2 expression in T47D cells exposed to 50 ng/mL of IL-6 for indicated times; (**B**,**C**) Wound healing assay of the migration ability of T47D cells in the presence or absence of IL-6. Data are expressed as mean ± SD. ** represents *p* < 0.01 as determined by Two way ANOVA.

An elevated activation of IL-6 signaling has been observed in many types of cancer [25,26,27]. Shp2 was reported to be involved in the Ras/Erk1/2 signaling pathway, triggered by IL-6 [28,29]; however, the detailed effect of Shp2 on IL-6 signaling-promoted cancer development and progression, is largely undefined. In the current study, the human breast cancer cell line T47D was treated with exogenous IL-6, for 72 h. As shown in Figure 1A, IL-6 induced a significant increase in N-cadherin, Vimentin, Erk1/2, and Shp2 phosphorylation, when compared with those in untreated cells. Interestingly, IL-6 also led to a notable downregulation of E-cadherin expression (Figure 1A). Consistently, IL-6 induced an apparent reduction of E-cadherin, and upregulation of Vimentin and N-cadherin (Supplementary Figure S1A). Its cell migration ability was also examined in the presence and absence of IL-6 in the culture medium, for 24 h. As shown in Figure 1B,C, a scratch assay showed that the migration distance in IL-6-treated cells was significantly longer than that in the control cells (Figure 1B,C) (*p* < 0.01). Likewise, exogenous IL-6 treatment also induces a significant increase in a cell’s invasive ability in MDA-MB-468 breast cancer cells (Figure S1B). Collectively, these data suggest that IL-6 may enhance the migration ability of breast cancer cells.

### 2.2. Stable Expression of IL-6 in T47D Cells Induces EMT In Vitro

**Figure 2:**
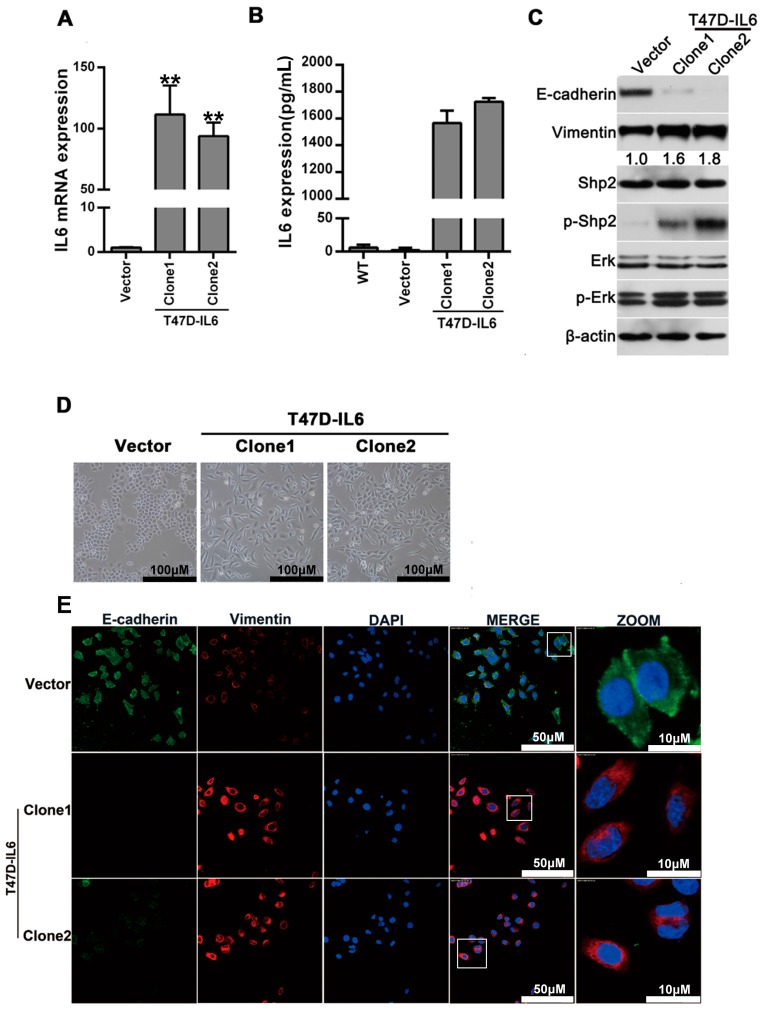

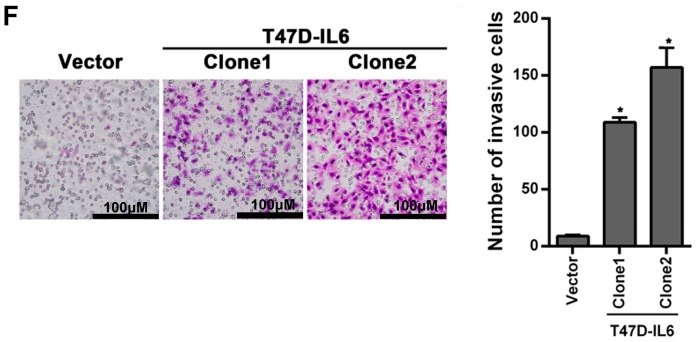
IL-6 overexpression can induce EMT in breast cancer cells. (**A**) Quantitative PCR analysis of the expression levels of IL-6 mRNA in the vector control and two IL-6-stable expression clones (*p* < 0.001); (**B**) The enzyme linked immunosorbent assay (ELISA) analysis of the secreted IL-6 protein level in the cell culture supernatant from the control and two stable clones; (**C**) Western blot analysis of the expression of E-cadherin, Vimentin, total and phosphorylated Erk1/2, total and phosphorylated Shp2 in cell lysates from the control, and IL-6-expressing T47D cells; (**D**) Stable expressing IL-6 induces a significant cell morphological change in normal culture condition; (**E**) The expression of E-cadherin and Vimentin in the vector control and IL-6-expressing T47D cells was examined by using immunofluorescence staining method; Squares: the areas which are chosen to magnify. (**F**) Transwell analysis of cell invasive ability. 1 × 10^6^ cells of the vector control, clone 1, and clone 2 groups were seeded and incubated with fetal bovine serum(FBS)-free Roswell Park Memorial Institute (RPMI)-1640 medium for 24 h in transwell chambers. The lower chamber was filled with RPMI-1640 containing 10% FBS. The cells that invaded through the membrane were fixed with methyl alcohol, stained, and counted using a light microscope. The number of cells was counted in five random microscopic fields. Data are expressed as mean ± SD. * means *p* < 0.05 as determined by One way ANOVA. ** represents *p* < 0.01.

To determine the effect of long-term IL-6 exposure on the migration and invasion capacities of breast cancer cells, T47D cells were infected with IL-6-expressing lentivirus, and two IL-6-stable expression clones were selected for analysis. As shown in Figure 2A, compared to that in the vector-infected cells, the expression level of IL-6 mRNA in the two stable clones increased by approximately 100-fold, as measured through the quantitative PCR assay. Consistently, the enzyme linked immunosorbent assay (ELISA) also showed that the level of secreted IL-6 protein in the cell culture supernatant from the two stable clones was significantly higher than that in the control cells (Figure 2B). The results of the Western blot analysis also showed a marked reduction in E-cadherin, and an increase in Vimentin expression in the T47D cells with IL-6-stable expression. This result supports an EMT signature induced by IL-6. In addition, the expression of phosphorylated Erk1/2 and Shp2 increased in cells with IL-6-stable expression, indicating that the activation of Erk1/2 pathways was triggered by IL-6 (Figure 2C). The cell morphology of the two clones with IL-6-stable expression displayed a significant disassociation of cell-cell junction, and became spindle-like in shape (Figure 2D). Accordingly, the immunofluorescence assay also indicated an obvious decrease in E-cadherin, and an increase in Vimentin expression in IL-6-stable expression cells (Figure 2E). Moreover, the transwell-based assay demonstrated that the IL-6-expressing T47D cells exhibited a notable enhancement in the cells’ invasive ability, compared to the vector-expressing cells (Figure 2F) (*p* < 0.05). Collectively, these results suggest that long-term exposure of breast cancer cells to IL-6 can induce an EMT phenotype.

### 2.3. Overexpression of IL-6 Promotes Tumor Metastasis In Vivo

**Figure 3:**
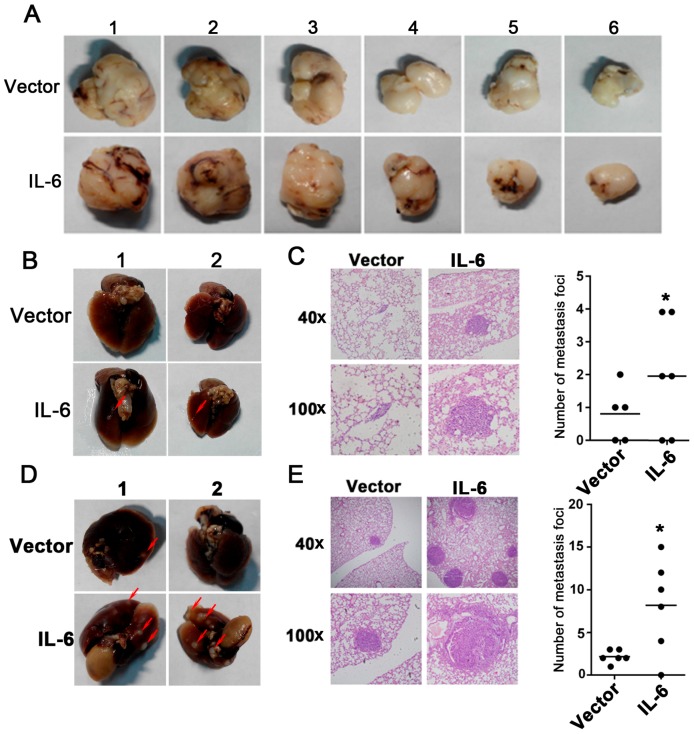
Overexpression of IL-6 increases tumor metastasis in vivo. (**A**) Representative image showing subcutaneous tumors from the vector (upper panel) and IL-6 overexpression (lower panel) groups; (**B**) Representative image of tumor foci on the mice lung surface; Red arrows: tumor foci on the mice lung surface; (**C**) H & E staining of mice lung slices shows that IL-6 overexpression increased the metastatic capacity of breast cancer cells in vivo. The number of metastasis foci in the IL-6 overexpression group is more than that in the vector group, * represents *p* < 0.05; (**D**) Representative image shows the tumor foci on the lung surface of mice by tail vein injection; Red arrows: tumor foci on the mice lung surface; (**E**) Using the tail vein injection method, H & E staining of the lung slices shows that IL-6 overexpression in tumors increased the metastatic capacity in vivo. The number of metastasis foci observed under a microscope shows that the IL-6 overexpression group has more foci than the vector group, *p* < 0.05.

We have already demonstrated that the stable expression of IL-6 in T47D cells induces EMT in vitro. Next, to test whether the overexpression of IL-6 promotes tumor metastasis in vivo, we established a tumor xenograft model, via the subcutaneous injection of the vector control or cells with IL-6 overexpression. Apparent subcutaneous tumor formation was observed 15 d after the tumor cell implantation in each group. The tumors were removed through a surgical operation five weeks after the inoculations. No significant differences in tumor volume and tumor weight were observed between the vector control group, and the IL-6 overexpression group (Figure 3A). After tumor removal surgery and another 50 days, the mice were anesthetized and sacrificed. The lungs of the mice were analyzed through H & E staining. As shown in Figure 3B, the lung metastasis foci in the IL-6 overexpression group had increased, compared to those in the vector control group (Figure 3B,C) (*p* < 0.05).

To further verify the influence of IL-6 on tumor metastasis, the vector control or IL-6-overexpression T47D cells were injected into the tail vein of mice. Metastatic tumors in the lung were detected five weeks after intravenous injection. As shown in Figure 3D, more metastatic foci were observed in the IL-6 overexpression group than in the vector group, on the surface of the lungs (Figure 3D). Figure 3E shows that H & E staining of lung tissue section slides was also performed, to evaluate the metastatic ability of tumor cells. More metastasis foci on the lung existed in the IL-6 overexpression group, than in the vector control group (Figure 3E) (*p* < 0.05). These findings indicate that the overexpression of IL-6 promotes breast cancer metastasis in vivo.

### 2.4. Knockdown of Shp2 Decreases Invasion and Migration Capacities In Vitro

**Figure 4:**
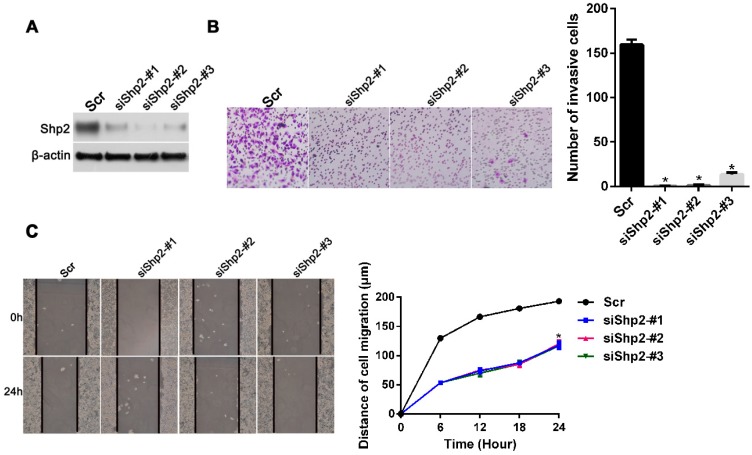
Knockdown of Shp2 decreases the invasion and migration capacities induced by IL-6 in vitro. (**A**) Western blot analysis of the expression of Shp2 in cell lysates from T47D cells transfected with negative control and Shp2 specific siRNAs. A scrambled (Scr) sequence was used as a negative control for siRNA transfection, which defined as “Scr”; (**B**) Scr and Shp2 knockdown cells were seeded and coated with Matrigel in the transwell chamber, as described in Methods. Images are representative of cells adhering to the lower chamber after the invasive process. Invading cells per filed in the lower chamber were counted (40×). Data are expressed as mean ± SD from five independent fields, * represents *p* < 0.05; (**C**) Wound healing assay of control and Shp2 knockdown cells. Cell images were obtained immediately (0 h), 6, 18, and 24 h later. The distance shows that Shp2 knockdown resulted in reduced cell migration ability, *p* < 0.05.

To determine whether Shp2 is involved in regulating the invasion and migration of breast cancer cells induced by IL-6, we knocked down Shp2 by using a specific siRNA in the T47D cell line. As expected, the expression of Shp2 decreased significantly in the three Shp2 siRNA transfected cells (Figure 4A). As measured through the transwell assay, Shp2 depletion in the T47D cells significantly reduced the number of cells that invaded the Matrigel-coated upper chamber (Figure 4B) (*p* < 0.05). Moreover, as shown in Figure 4C, the cells with Shp2 downregulation closed the wound scratch more slowly than the negative control cells did (Figure 4C) (*p* < 0.05). Collectively, these results indicate that Shp2 plays an important role in the invasion and migration of breast cancer cells.

### 2.5. Knockdown of Shp2 Attenuated EMT Induced by IL-6 In Vitro

**Figure 5:**
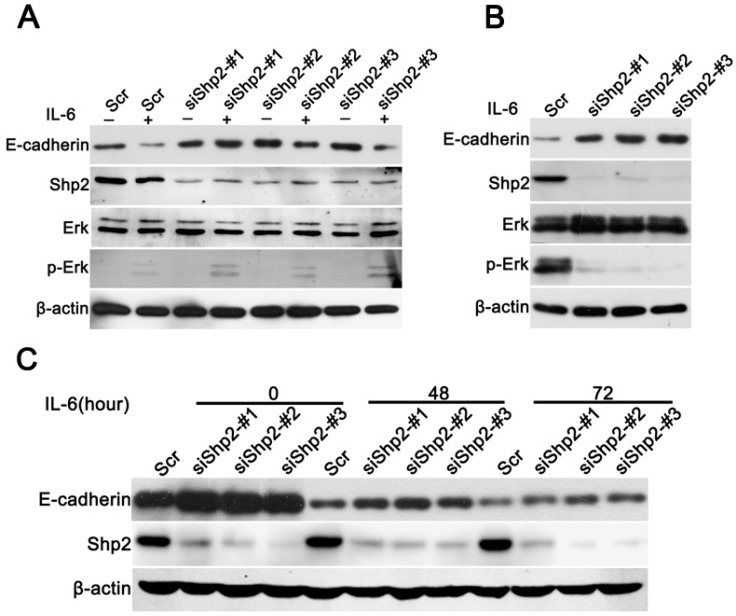
Knockdown of Shp2 attenuates IL-6-induced EMT in vitro. (**A**) Western blot analysis of the effect of Shp2 knockdown on the IL-6 signal pathway. T47D cell exhibited an MET phenotype characterized by an increase in E-cadherin when Shp2 was downregulated. After 15 min of stimulation of IL-6, the expression of Shp2 did not significantly affect Erk activity; (**B**) Knockdown of Shp2 attenuated the IL-6-triggered phosphorylation of ERK. Scr and Shp2 knockdown cells were starved overnight and treated with IL-6 for 72 h. The expression of Erk and phosphorylated Erk was analyzed by the Western blot method; (**C**) Knockdown of Shp2 attenuated the IL-6-induced downregulation of E-cadherin. Scr and Shp2 knockdown cells were treated with IL-6 for 0, 48, and 72 h, and the expression of E-cadherin and Shp2 was analyzed by the Western blot method.

We previously found that IL-6 induces EMT, and that the knockdown of Shp2 decreases invasion and migration capacities in the T47D cell line. Next, we verified the role of Shp2 in IL-6-induced EMT. As shown in Figure 5A, knockdown of Shp2 in T47D cells induced an MET phenotype characterized by an E-cadherin increase, compared to that in Scr cells. Moreover, Shp2 downregulation inhibited the IL-6-induced phosphorylation of Erk1/2 (Figure 5A,B). Knockdown of Shp2 attenuated the IL-6-induced downregulation of E-cadherin (Figure 5C). In conclusion, Shp2 is critical for IL-6-induced EMT in the T47D cell line.

### 2.6. Phosphatase Activity and Tyrosine Phosphorylation of Shp2 Regulate IL-6-Induced EMT

**Figure 6:**
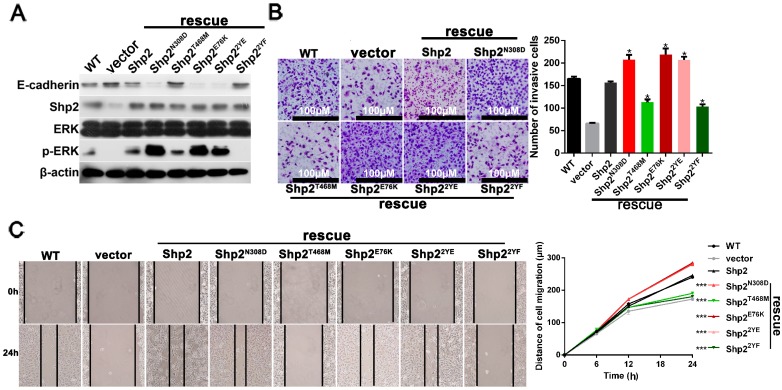
Both the phosphatase activity and tyrosine phosphorylation of Shp2 regulate EMT induced by IL-6. (**A**) Expression of active forms of Shp2 mutants or tyrosine phospho-mimicking Shp2 mutant in Shp2-silencing cells, resulted in upregulation of Erk phosphorylation and downregulation of E-cadherin. Shp2-knockdown T47D cells were infected with lentivirus expressing the vector control, wild-type Shp2, or different mutant forms of Shp2. The cells were stimulated with IL-6 for 72 h, and total proteins were subjected to Western blot analysis with antibodies against E-cadherin, Shp2, Erk, p-Erk, and β-Actin. “WT” means wild type T47D cells; (**B**) In the transwell assay, cells rescued by Shp2^N308D^, Shp2^E76K^, and Shp2^2YE^ exhibited a more powerful invasion ability than cells with wild-type Shp2. The Shp2^T468M^ and Shp2^2YF^ cells showed a weaker invasion ability than the wild-type Shp2. * means *p* < 0.001; (**C**) Wound healing assay of T47D cells rescued the expression of the vector control, wild-type Shp2, or different forms of Shp2 mutants. According to the result of the transwell assay above, the cells rescued by Shp2^N308D^, Shp2^E76K^, and Shp2^2YE^ exhibited a strong migration ability, whereas the Shp2^T468M^ and Shp2^2YF^ cells exhibited the opposite. *** means *p* < 0.001.

We have indicated that Shp2 plays an important role in IL-6-induced EMT. Given that Shp2 is a well-known tyrosine phosphatase, we aimed to determine whether the phosphatase activity of Shp2 is required for IL-6-induced EMT. Wild-type Shp2, the vector control (PCDH), two Shp2 gain-of-function mutants (Shp2^N308D^ and Shp2^E76K^), and a phosphatase-deficient mutant (Shp2^T468M^), were constructed and introduced into Shp2-depleted breast cancer cells. As shown in Figure 6A, the expression of the two gain-of-function Shp2 mutants, but not the Shp2^T468M^ mutant, enhanced Erk phosphorylation, and resulted in a significant decrease in E-cadherin, when compared with that in the wild-type Shp2 cells. Several reports indicate that the tyrosine phosphorylation of Shp2 at Tyr542 and Tyr580 is the most important post-translational modification [4]. To determine if tyrosine phosphorylation of Shp2 regulates IL-6-induced EMT in breast cancer cells, a phospho-mimicking mutant form of Shp2 (Shp2^2YE^) and a phospho-deficient mutant form of Shp2 (Shp2^2YF^), were introduced into the Shp2 knockdown cells. As shown in Figure 6A, the expression of phospho-mimicking Shp2, but not the phospho-deficient Shp2 mutant, led to a significant enhancement of Erk phosphorylation, and the downregulation of E-cadherin, when compared with that in the wild-type Shp2 cells. We also found that the re-expression of Shp2^N308D^, Shp2^E76K^, and Shp2^2YE^ mutants, but not Shp2^T468M^ and Shp2^2YF^ mutants, rescued the cell invasive ability, as measured by the transwell-based assay (Figure 6B), *p* < 0.001. The wound healing assay also showed that cells expressing Shp2^N308D^, Shp2^E76K^, or Shp2^2YE^ mutants, closed the wound more rapidly than the cells carrying the wild-type Shp2; The cells with Shp2^T468M^ and Shp2^2YF^ closed the wound more slowly than the wild-type Shp2 cells (Figure 6C), *p* < 0.001. In summary, these results suggest that phosphatase activity and the tyrosine phosphorylation of Shp2, are both required for IL-6-induced EMT.

## 3. Discussion

Persistent activation of the IL-6-signaling pathway has been observed in many types of tumors, and contributes to carcinogenesis and cancer progression [30,31]. The binding of IL-6 to its receptor canonically activates the JAK-STAT3 signal pathway. IL-6 also activates Ras/Erk1/2 [32]. Tyrosine phosphatase Shp2 functions as a pivotal linker between the IL-6 receptor and the Ras/Erk1/2 pathway [29]. Abnormal expression of Shp2 has been implicated in many human malignancies [33,34]. However, the accurate function of Shp2 in IL-6-signaling-promoted cancer development and progression, is largely undefined. The present study showed that exogenous and endogenous IL-6 can enhance breast cancer invasion and migration, through the promotion of EMT. IL-6 also induces the activation of Erk1/2 and the phosphorylation of Shp2. The results demonstrated that Shp2 is required for the IL-6-induced EMT of breast cancer cells, and both the phosphatase activity of Shp2, and its tyrosine phosphorylation, are necessary for EMT triggered by IL-6. The findings uncover an important function that links Shp2 to IL-6-promoted breast cancer progression.

IL-6 signaling was traditionally defined as a pivotal mediator in the regulation of the immune response, by inducing B cell differentiation. In addition, IL-6 signaling is involved in the regulation of inflammation, hematopoiesis, and embryonic development [35,36]. In a tumor microenvironment, IL-6 is one of the major cytokines that can be secreted by tumor and stromal cells [37]. Activation of IL-6 signaling also promotes oncogenesis, by regulating cancer cell survival, proliferation, apoptosis, angiogenesis, and drug resistance [38]. Moreover, IL-6 promotes the migration and invasion of multiple tumor cells, via the induction of EMT [39]. For example, the overexpression of IL-6 enhances the invasion and migration of gallbladder cancer cells, by stimulating EMT [40]. In this study, we found that exogenous IL-6 induced a significant downregulation of E-cadherin, and an enhancement of migration in the breast cancer cell line T47D. Additionally, the overexpression of IL-6 in T47D promoted EMT and metastatic ability, both in vitro and in vivo. Consistently, another study revealed that the elevation of IL-6 promotes the mesenchymal phenotype in the MCF-7 cell line, and that mice with MCF-7^IL−6^-transplanted tumors exhibit a higher invasive and metastatic ability than control group mice [24,41]. These data suggest that EMT induction may be the major mechanism by which IL-6 promotes breast cancer metastasis.

Shp2 is the first identified oncoprotein with protein tyrosine activity [42]. The well-known function of Shp2 is its positive role in regulating receptor tyrosine kinase signaling, which is critical for the initiation and progression of many types of cancer. In addition to mediating growth factor receptor signal transduction, Shp2 is also involved in other signaling pathways, such as IL-6 signaling [21]. However, the detailed function of Shp2 in IL-6-signaling-promoted breast cancer aggravation, remains uncertain. In the current study, the deletion of Shp2-inhibited Erk phosphorylation and theIL-6-induced enhancement of cell migration and invasion, indicates that Shp2 may also act as a positive regulator in IL-6-signaling-mediated cancer progression. Consistent with this observation, other studies have reported that Shp2 contributes to the migration, invasion, and metastasis of breast cancer cells [43,44]. We also found that Shp2 silencing attenuated the IL-6-induced downregulation of E-cadherin, a hall marker of EMT. Several recent studies have similarly shown that Shp2 is required for TGF-β, EGF, or PDGFRα-driven EMT, in various types of cancers [29]. These results suggest that the requirement of Shp2 may be a common molecular mechanism for various signaling-triggered EMT. Interestingly, a recent study has provided evidence to support this possibility. This study showed that Shp2 is a binding protein of PAR3, a critical component of the polarity protein complex, which consists of PAR3, PAR6, and aPKC [45]. Shp2 dephosphorylates PAR3, and thereby reduces the formation of this protein complex, leading to the disassociation of cell-cell junctions, loss of cell polarity, and enhancement of EMT [45]. Collectively, these data demonstrate that Shp2 can promote EMT through multiple mechanisms in different types of cancers.

Although the promoting role of Shp2 in cancer development and progression has been well accepted, the effect of its phosphatase activity on cancer cell migration, invasion, and EMT, is largely unclear, particularly in solid tumors. We showed that the rescued expression of two Shp2 GOF mutants, but not the LOF mutant Shp2^T468M^, resulted in a significant elevation of ERK phosphorylation, and a reduction of E-cadherin expression. Meanwhile, the re-expression of Shp2 GOF mutants in Shp2 knockdown cells, enhanced cell migration and invasion abilities, when compared with the wild-type Shp2. Consistently, two recent studies have reported that the phosphatase-active mutant of Shp2 facilitates EMT, whereas the expression of the phosphatase-dead form inhibits EMT in lung and oral cancer cells [46,47]. These data suggest that the phosphatase activity is required for Shp2 to regulate EMT in cancer cells. In addition, another report demonstrated that the inhibition of Shp2 phosphatase activity using selective inhibitors, blocks TGF-β1-stimulated EMT in lung cells, or HGF-induced EMT in pancreatic adenocarcinoma cells [15], which further confirms the requirement of Shp2 phosphatase activity for EMT in cancer cells. Interestingly, we found that re-expression of the phospho-mimicking mutant Shp2 (Shp2^2YE^), but not the phospho-deficient mutant form (Shp2^2YF^), rescued IL-6-induced EMT. This result indicates that tyrosine phosphorylation, an important post-translational modification for Shp2, is also critical for the EMT of cancer cells. A possible explanation is that the tyrosine phosphorylation of Shp2 promotes its phosphatase activity [48]. Alternatively, these modifications may be essential for the Shp2 function in regulating Erk phosphorylation [49]. Altogether, our results suggest that both the phosphatase activity of Shp2 and its tyrosine phosphorylation, are pivotal for IL-6-triggered EMT.

## 4. Materials and Methods

### 4.1. Cell lines and Culture

The human breast cancer cell line T47D and renal embryonic cell line 293T were purchased from American Type Culture Collection (ATCC, Manassas, VA, USA). The T47D cells were routinely maintained in RPMI-1640 medium (Hyclone, Logan, UT, USA), supplemented with 10% fetal bovine serum (FBS; Hyclone, Logan, UT, USA). The 293T cells were maintained in DMEM (Hyclone, Logan, UT, USA), supplemented with 10% FBS. All cell lines were stored in humidified incubators at 37 °C and with 5% CO_2_ in the laboratory. IL-6 (Peprotech, Rocky Hill, NJ, USA) was added to all cultures at final concentrations of 50 ng/mL, in RPMI-1640 medium containing 10% FBS.

### 4.2. siRNA Transfection

Three Shp2 specific Stealth^TM^ siRNA, targeting the non-coding region of Shp2 (siShp2 sequence 1: 5′-GACAUCCCUCUUUGCCUCAUAUGUU-3′; siShp2 sequence 2: 5′-CGAGGUCAGCAAACUAUCAUGUUCU-3′; siShp2 sequence 3: 5′-UACAGAUCCUAACAAAGGCAUCCUG-3′), and a negative control siRNA (Scr), were purchased from Invitrogen Company. Transfection was performed using the Lipofectamine 2000 (Invitrogen, Carlsbad, CA, USA) method, according to the manufacturer’s instructions. After transfection for 72 h, the cells were harvested for further analysis.

### 4.3. Vector Construction, Transfection, and Stable Cell Line Acquisition

The total RNA acquired from the breast cancer cell line MDA-MB-231 was reverse transcribed, and the coding region of the IL-6 gene was amplified through a polymerase chain reaction (PCR), with the following specific primers: Upper: CGGAATTCATGAACTCCTTCTCCACAAGCGCC; Lower: GAGGATCCCTACATTTGCCGAAGAGCCC. The coding region of IL-6 was cloned into a lentiviral vector PCDH in the BamH1 and EcoR1 cloning sites. The Lentivirus producer cell line 293T cells were co-transfected with the lentiviral vector and virus packaging plasmids, by using Lipofectamine 2000, according to the manufacturer’s instruction. Virus supernatants were used to infect the T47D cells. Then, the cell lines stably expressing the control or IL-6, were selected and maintained in 1 µg/mL puromycin (Sigma, St. Louis, MO, USA), and two different cell clones were screened for further analysis. Wild type Shp2 (Shp2^WT^) was amplified through PCR, with the following specific primers: Uppper: 5′-CGGAATTCATGACATCGCGGAGATGGTTTC-3′ lower: 5′-GAGGATCCTCATCTGAAACTTTTCTGCTGT-3′, and cloned into a lentiviral vector PCDH in the BamH I (Thermo Scientific, Waltham, MA, USA) and EcoR I (Thermo) cloning sites. Shp2 mutants were generated by site-directed mutagenesis, using a Quick-change mutagenesis kit (Stratagene, La Jolla, CA, USA). The mutation of GAA (E) to AAA (K) at the amino acid 76 encoding position, and the mutation of AAT (N) to GAT (D) at the amino acid 308 encoding position, generated two Shp2 constitutively phosphatase active mutants. The mutation of ACG (T) to ATG (M) at the amino acid 468 encoding positionm generated a Shp2 phosphatase inactive mutant. The double mutations of TAT(Y) to TTT (F) at the amino acid 542 and 580 encoding positions, generated a Shp2 phosphor-tyrosine deficiency mutant. The double mutations of TAT(Y) to GAA (E) at the amino acid 542 and 580 encoding positions, generated a Shp2 phosphor-tyrosine mimicking mutant. The recombination plasmids were verified by DNA sequencing.

### 4.4. Western Blot Analysis

Western blotting was performed as described previously [50]. The antibodies used in this study included the following: mouse monoclonal antibodies against β-actin (1:1000), gp130 (1:500) from Santa Cruz Biotechnology, E-cadherin (1:3000) from BD Biosciences (San Jose, CA, USA), rabbit monoclonal antibodies against SHP2 (1:1000) from Santa Cruz Biotechnology, *N*-cadherin (1:1000), Vimentin (1:1000), Erk1/2 (1:1000), p-Erk (1:1000), and p-SHP2 (1:500) from Cell Signaling Technology. All primary antibodies were diluted with 5% BSA in tris buffered saline tween-20 (TBST), and the membranes were incubated at 4 °C overnight. The membranes were washed with TBST three times and then incubated with HRP-linked secondary antibodies (Santa Cruz, CA, USA) for 1 h, at room temperature. All Western blots were detected by electrochemiluminescence (Amersham Pharmacia Biotech, Aylesbury, UK). β-actin was used as the internal control.

### 4.5. Wound Healing Assay

A wound healing assay was performed to assess the migration ability of the cell lines. The cells were cultured to 100% confluence, in a six-well cell culture plate. A scratch was created with a 10 μL pipette tip. The culture was washed with PBS to remove cell debris, and incubated in a medium without FBS, at 37 °C. The migration distances were recorded at 0, 6, 12, 18, and 24 h. For IL-6-stimulated migration, cells were starved overnight, and wounds were created in the same manner as that mentioned above. The cells were re-fed with serum-free media and treated with 50 ng/mL of IL-6 for 24 h, or were left untreated. Images of the wounds were captured at different time points. The Olympus Microsuite software (Tokyo, Japan) was used to estimate the cell-free area of the wounds (40×). All assays were performed in triplicate.

### 4.6. Transwell Invasion Assay

A cell invasion assay was performed using a transwell insert with 8 μm filter pores (Corning Costar Corporation). The upper side of the chamber was covered with 240 µL of 0.25 mg/mL Matrigel (BD Bioscience) at 37 °C, for 1 h. After washing with PBS, 200 µL of cells (1 × 10^6^ cells/mL) was loaded into the upper chamber. The lower chamber was filled with 600 µL of RPMI-1640, containing 10% FBS. After incubation at 37 °C for 24 h, the cells that invaded through the membrane, were fixed and stained with the Three-Step Stain Set (Thermo Scientific, Waltham, MA, USA). Invasive cells were quantified by recording five random microscopic fields and expressed as a number of invasive cells. All assays were performed in triplicate.

### 4.7. Tumor Metastasis Experiment In Vivo

The experimental protocol was approved by the Animal Ethical and Welfare Committee of Tianjin Medical University Cancer Institute and Hospital (Project identification code: 2016090; Date: 10 February 2016). Six-week-old female immunodeficient mice (Vital Laboratory Animal Center, Beijing, China) were used as animal models. A total of 24 mice were divided into four groups (A, B, C, and D), through random sampling. In groups A and B, 2 × 10^6^ control or IL-6-expressing T47D cells, were injected into the subcutaneous fat of the mice. In groups C and D, 1 × 10^6^ cells were injected into the tail vein of mice. For groups A and B, five weeks after cell injection, the tumors were removed via a surgical operation; the mice were allowed to grow for another 50 d, and then sacrificed by an intraperitoneal injection of overdose pentobarbital sodium. The mice in groups C and D were sacrificed five weeks after cell injection. Lung tissues were anatomized and fixed in 4% paraformaldehyde. The tumor nodules on the surface of the lung lobe were counted, and the numbers were statistically analyzed. The lung tissues were embedded in paraffin and stained with hematoxylin–eosin (H & E) for microscopy observation.

### 4.8. Statistical Analysis

Data were expressed as mean ± SD. The differences among groups were evaluated through one-way or two-way ANOVA, using the GraphPad Prism 7.00 software (Graphpad Software, La Jolla, CA, USA). *p*-values less than 0.05 (two-tailed) were considered statistically significant. * indicates that the *p*-value is less than 0.05, ** means *p*-value is less than 0.01.

## 5. Conclusions

In conclusion, our study showed that IL-6 can enhance breast cancer invasion and migration, through the promotion of EMT. Shp2 is essential for the IL-6-induced EMT of breast cancer cells, and both the phosphatase activity of Shp2 and its tyrosine phosphorylation, are necessary for the EMT triggered by IL-6.

## Supplementary Materials

Supplementary materials can be found at www.mdpi.com/1422-0067/18/2/395/s1.

## Abbreviations

EMT	Epithelial mesenchymal transition
Shp2	Src-homology 2 domain-containing tyrosine phosphatase 2
PTP	Protein tyrosine phosphatase
