# Supplementary material for: Shp2 Plays a Critical Role in IL-6-Induced EMT in Breast Cancer Cells
Source: Int J Mol Sci. 2017 Feb 13;18(2):395. doi: 10.3390/ijms18020395 (PMC5343930; doi:10.3390/ijms18020395)
Supplement: Supplementary file 1 [file ijms-18-00395-s001.pdf]

Supplementary Materials: Shp2 Plays a Critical Role in IL-6-Induced EMT in Breast Cancer Cells

Xuan Sun, Jie Zhang, Zhiyong Wang, Wei Ji, Ran Tian, Fei Zhang and Ruifang Niu

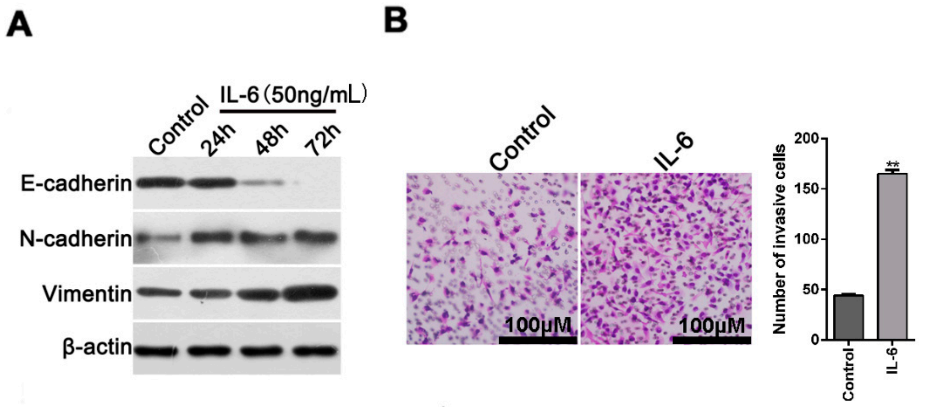

Figure S1. Exogenous IL-6 treatment induces a significant increase in cell invasive ability in vitro. (**A**) Western blot analysis of E-cadherin, N-cadherin, Vimentin expression in MDA-MB-468 cells exposed to 50 ng/mL of IL-6 for 24, 48 and 72 h; (**B**) Transwell assay show the invasive ability of MDA-MB-468 cells in the presence or absence of IL-6. Data are expressed as mean ± SD from five independent fields, ** $p < 0.01$.
